# Qualitative Approach to Comparative Exposure in Alternatives Assessment

**DOI:** 10.1002/ieam.4070

**Published:** 2018-07-19

**Authors:** William Greggs, Thomas Burns, Peter Egeghy, Michelle R Embry, Peter Fantke, Bonnie Gaborek, Lauren Heine, Olivier Jolliet, Carolyn Lee, Derek Muir, Kathy Plotzke, Joseph Rinkevich, Neha Sunger, Jennifer Y Tanir, Margaret Whittaker

**Affiliations:** ^1^ Soleil Consulting Sanibel Florida USA; ^2^ Novozymes, Research Triangle Park North Carolina USA; ^3^ US Environmental Protection Agency Durham North Carolina; ^4^ Health and Environmental Sciences Institute Washington DC USA; ^5^ Technical University of Denmark Kongens Lyngby Denmark; ^6^ DuPont Haskell Global Centers for Health and Environmental Sciences Newark Delaware USA; ^7^ Northwest Green Chemistry Spokane Washington USA; ^8^ University of Michigan Ann Arbor Michigan USA; ^9^ ExxonMobil Biomedical Sciences Annandale New Jersey USA; ^10^ Environment and Climate Change Canada Burlington Ontario; ^11^ Dow Chemical Midland Michigan USA; ^12^ SciVera Charlottesville Virginia USA; ^13^ West Chester University West Chester Pennsylvania USA; ^14^ ToxServices Washington DC USA

**Keywords:** Exposure assessment, Consumer products, Data selection, Parameter relevance, Chemical substitution

## Abstract

Most alternatives assessments (AAs) published to date are largely hazard‐based rankings, thereby ignoring potential differences in human and/or ecosystem exposures; as such, they may not represent a fully informed consideration of the advantages and disadvantages of possible alternatives. Building on the 2014 US National Academy of Sciences recommendations to improve AA decisions by including comparative exposure assessment into AAs, the Health and Environmental Sciences Institute's (HESI) Sustainable Chemical Alternatives Technical Committee, which comprises scientists from academia, industry, government, and nonprofit organizations, developed a qualitative comparative exposure approach. Conducting such a comparison can screen for alternatives that are expected to have a higher or different routes of human or environmental exposure potential, which together with consideration of the hazard assessment, could trigger a higher tiered, more quantitative exposure assessment on the alternatives being considered, minimizing the likelihood of regrettable substitution. This article outlines an approach for including chemical ingredient‐ and product‐related exposure information in a qualitative comparison, including ingredient and product‐related parameters. A classification approach was developed for ingredient and product parameters to support comparisons between alternatives as well as a methodology to address exposure parameter relevance and data quality. The ingredient parameters include a range of physicochemical properties that can impact routes and magnitude of exposure, whereas the product parameters include aspects such as product‐specific exposure pathways, use information, accessibility, and disposal. Two case studies are used to demonstrate the application of the methodology. Key learnings and future research needs are summarized. *Integr Environ Assess Manag* 2018;00:000–000. © 2018 The Authors. *Integrated Environmental Assessment and Management* published by Wiley Periodicals, Inc. on behalf of Society of Environmental Toxicology & Chemistry (SETAC)

## Key Points

- Alternatives Assessment (AA) describes the approach to identify, compare, and select safer and more sustainable alternatives to chemicals of concern.
- Alternatives Assessments have often been hazard‐based rankings used to substitute individual ingredients and may not represent a fully informed consideration of advantages or disadvantages of possible alternative solutions.
- Chemical alternatives that may have a higher or different route of exposure potential (human or environmental) can be identified via a qualitative exposure approach, which could trigger a higher tiered, more quantitative assessment to minimize the likelihood of regrettable substitution.
- This work outlines a classification approach for including chemical ingredient‐ and product‐related exposure information to support comparisons between alternatives in a qualitative manner using 2 case studies.

## EDITOR'S NOTE

This article is part of the special series “Advances in Methods and Practice of Alternatives Assessment.” Alternatives Assessment is an evolving approach for identifying, comparing, and selecting safer alternatives to chemicals of concern based on their hazards, comparative exposure, performance, and economic viability. While alternatives assessment frameworks have existed for over 20 years, the 2014 publication of the National Research Council's *Framework to Guide the Selection of Chemical Alternatives* firmly established the field. Since then, scientists from academia, government, business, and nonprofits have convened on numerous occasions, including at SETAC meetings, to identify methodological and practice needs for the field as well as form a new multidisciplinary society to support a growing community of practitioners—the Association for the Advancement of Alternatives Assessment. The articles in this series arose from numerous multistakeholder efforts and describe methodological developments in alternatives assessment, lessons from practical application, an assessment of current gaps in methods and practice, and outcomes of a 2018 international symposium on alternatives assessment.

## INTRODUCTION

Alternative Assessment (AA) describes the approach to identify, compare, and ultimately select safer alternatives to chemicals of concern (MA TURI 2013). The overall goal of AA is to support informed decisions regarding advantages and disadvantages of different alternatives to harmful chemicals in various product applications (NRC 2014b). Most AAs published to date are not truly AAs. Rather, they are largely hazard‐based rankings of alternatives, which usually are restricted to substitute individual ingredients; as such, they may not represent a fully informed consideration of the advantages and disadvantages of possible alternative solutions, including chemicals, materials, technologies, or behavioral changes (Fantke et al. 2015). Sustainable chemistry, as defined by the Organisation for Economic Co‐operation and Development (OECD), is “…a scientific concept that seeks to improve the efficiency with which natural resources are used to meet human needs for chemical products and services” (OECD 2012). With an assessment goal of identifying alternatives that are safer and more sustainable than the original ingredient, other attributes beyond hazard are also important, including exposure, life cycle impacts, material or product performance, costs, and social responsibility, as outlined in guidance frameworks for AA developed by the US National Academy of Sciences (NAS) and 19 others (reviewed by Jacobs et al. 2016). Of these aspects, we focus on the exposure component as a starting point to improve existing AA practice with emphasis on chemical substitutes.

The NAS (2014b) report outlined 2 approaches for a comparative exposure assessment. The first (called “Path A”) is a quantitative approach, adapting existing models or developing new models and applying them to the reasonably foreseeable use and disposal scenarios for a product containing an ingredient and its potential alternatives. This path has been elaborated by Arnold et al. (2017), who described an approach to integrate quantitative exposure information into a risk‐based screening methodology for AA. The second (called “Path B”) is a property‐based approach, compiling and comparing physical and chemical properties that can be used to predict human exposure and environmental fate while considering the reasonably foreseeable use and disposal scenarios.

Building on these 2014 recommendations to improve AA decisions by including comparative exposure assessment, the Health and Environmental Sciences Institute (HESI) Sustainable Chemical Alternatives Technical Committee, which consists of scientists from academia, industry, government, and nonprofit organizations, developed a comparative exposure assessment procedure. This qualitative methodology follows the property‐based approach (Path B) from NAS (2014b). Conducting such a comparison can screen alternatives to understand whether they are expected to have a higher, lower, substantially equivalent, or different route of human or environmental exposure potential. If exposures are likely to be higher, different in route, or uncertain, the information should be considered in concert with hazard information to evaluate whether a higher tiered, more quantitative assessment is necessary, minimizing the likelihood of regrettable substitution.

The goal of the present study was to pilot the concept of integrating exposure information into the AA process, using a qualitative comparative approach based on evaluating a set of ingredient and product parameters. Case studies were selected to develop the comparative methodology and to understand the requirements and value of the effort. The target and alternative ingredients used in the case studies were selected from existing AAs that addressed hazard only.

## METHODOLOGY

**Figure 1:**
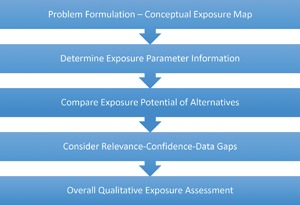
Qualitative comparative exposure assessment methodology for alternatives assessment.

Following the NAS recommendations, we developed a stepwise qualitative methodology (shown in Figure 1) to compare exposure profiles between a target ingredient in a product and any potential replacement. The process and rationale are described in the following sections. Starting with conceptual maps, we 1) define the scenario, 2) identify exposure‐relevant parameters for both ingredients and products, 3) define criteria for comparing the parameters for different alternatives, 4) outline an approach to assess the relevance of each parameter to a specific product as well as the confidence in the data being compared and any data gaps, and 5) finally, describe an approach to make conclusions about the overall assessment.

**Figure 2:**
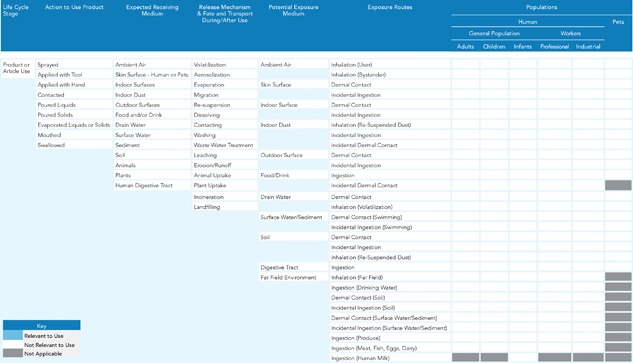
Generic conceptual map for human populations.

**Figure 3:**
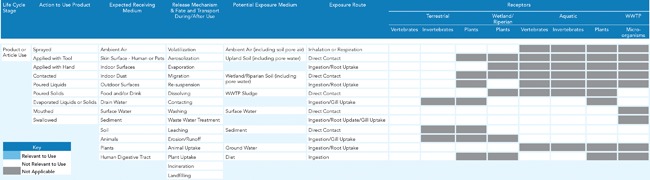
Generic conceptual map for ecological receptors.

### Problem formulation: Conceptual map

To focus the comparative exposure assessment for target and alternative ingredients in a product, a useful first step is to develop a conceptual map. Conceptual maps are representations of key relationships in a system and help understand or simulate the subject the map represents (USEPA 1992, 1998).

In the context of exposure assessment, conceptual maps help to identify all potentially relevant exposure pathways for all potentially exposed human and ecological populations based on the uses of the product. To achieve this for all possible product types, 2 separate generic conceptual maps were developed for human and ecological receptors, respectively (Figures 2 and 3). The first iteration of the conceptual map focuses on

- the action one must take to make the product work,
- the expected receiving medium or media of the product ingredients,
- the potential release mechanisms and fate and transport processes of the product ingredients during and after use, and
- the potential exposure medium or media and the potential exposure routes through which people, plants, and/or animals may come into contact with the product ingredients.

These generic maps enable the representation of likely exposure pathways for all possible product types in the market. To apply these maps to a specific product type, relevant items (boxes) in Figures 2 and 3 would be highlighted based on how the product is intended to be used and how the product may reasonably be misused. This highlighting is illustrated in the case studies (see Supplemental Data Document 2, Figures S2–S5) to demonstrate its application. Boxes without any highlighting (devoid of color or pattern) indicate that the item is not relevant to the use that is being evaluated. In addition, several boxes have been identified as not applicable to a particular exposure route, especially for some of the ecological receptors in Figure 3. Although careful deliberation was employed to identify receptors or populations usually not linked to certain exposure routes, professional judgment may deem these “not applicable” boxes as “applicable” for some product uses. Likewise, different individuals may highlight different boxes when completing the conceptual maps for a product use. Therefore, to minimize different conclusions, it is important that this work be performed by individuals with experience conducting exposure assessments and be reviewed by other experts.

The highlighted system of mapped boxes focuses and informs the next step of the qualitative exposure assessment, which is the collection of product parameter information and physical and chemical properties for each ingredient parameter. It may be possible to refine the conceptual maps where irrelevant release mechanisms, fate and transport processes, exposure routes, and exposed populations are removed. If further refinement is necessary, it is possible to develop quantitative maps that are able to track exposure pathways and receptors according to their contribution to overall exposure (Lanters and Fantke 2018). Our conceptual maps are not designed to weigh different mapped aspects but only to visualize them for reducing complexity and focusing the assessment.

### Exposure parameters and their importance

The general population is typically exposed to chemicals indirectly through environmental emissions and/or directly through use and contact with consumer products and other products such as furnishings and building materials (Jolliet et al. 2015). For example, ecological receptors are usually exposed only through the indirect pathways following use and disposal, although products that are applied to soil or used in agricultural situations could result in direct exposure. Inherent properties of substances not only define the physical and biological hazards of a chemical but also constitute an important component contributing to the potential for human and ecological exposure in the context of a given product use and disposal scenario.

For this comparative methodology, a total of 16 ingredient parameter categories (25 including subparameters) were identified, including both physical and chemical properties and characteristics of substances (Table 1).

**Table 1:** Ingredient parameters and classification

Nr	Ingredient parameter	Classification	Source of classification
1	SMILES	No classification system, expert consideration	USEPA 2013
2	Structure	No classification system, expert consideration	USEPA 2013
3	Vapor pressure	Phases (in mm Hg)	USEPA 2013
		Mostly vapor: >10^−4^	
		Vapor–particulate: 10^−5^ to 10^−7^	
		Solid: <10^−8^	
4	Solubility in water	Solubility (in mg/L)	USEPA 2013; 2015
		Very soluble: >10 000	
		Soluble: >1000–10 000	
		Moderate: >100–1000	
		Slightly soluble: >0.1–100	
		Negligible solubility: <0.1	
		Insoluble: <0.001	
5	Molecular weight	Low dermal absorption: >500 g/mol	OECD 2008
6	Particle attribute (size)	Likely to penetrate the alveolar region <10 µm	ACGIH 1993
		Likely to enter the nose or mouth and penetrate the tracheo‐alveolar region ≥10 and ≤100 µm	
		Not likely to be inhaled >100 µm	
		Inhalable fraction (in mg/kg)	ART (2016)
		Firm granules, flakes, or pellets: ≤100	
		Granules, flakes, or pellets: 100–500	
		Course dust: 501–2000	
		Fine dust: >2000–5000	
		Extremely fine and light powder: >5000	
7	Ambient physical state (melting point or boiling point)	Solid: melting point >25 °C	USEPA 2015
		Liquid: melting point <25 °C or boiling point >25 °C	
		Gas: boiling point <25 °C	
8	Bioavailability (skin permeability: log *K*_p_; human oral absorption: %)	Log *K*_p_: no known classification system; the lower the better	N/A
		Human oral absorption: no known classification system; the lower the better	
9	Octanol–water partition coefficient (log *K*_ow_)	Log *K*_ow_ value	
		Water‐soluble/bioavailable: <4	USEPA 2015
		Tendency to bioaccumulate: ≥4	
		Highly soluble in water: <1	USEPA 2013
		Not very soluble in water: >4	
		Not readily bioavailable: >8	
		Not bioavailable: >10	
10	Octanol–air partition coefficient (log *K*_oa_)	Log *K*_oa_ value	Kelly et al. 2007
		>6: strong association with lipid or organic surfaces. Not readily exhaled by air‐breathers	
		>6–<12 and >2–<9: chemicals highly bioaccumulative in the food chain to humans	
11	Soil sorption partition coefficient (log *K*_oc_)	Log *K*_oa_ value	USEPA 2013
		Very strong sorption, negligible migration: >4.5	
		Strong sorption, negligible to slow migration: 3.5–4.4	
		Moderate sorption, slow migration: 2.5–3.4	
		Low sorption, moderate migration: 1.5–2.4	
		Negligible sorption, rapid migration: <1.5	
12	Henry's Law constant	Henry's Law constant value	USEPA 2013
		Very volatile from water: >10^−1^	
		Volatile from water: 10^−1^ to 10^−3^	
		Moderately volatile: 10^−3^ to 10^−5^	
		Slightly volatile: 10^−5^ to 10^−7^	
		Nonvolatile: <10^−7^	
13	Bioaccumulation (BAF/BCF)	BAF (log BAF) or BCF (log BCF)	USEPA 2015
		Very high: >5000 (>3.7)	
		High: 5000 to 1000 (3.7 to 3)	
		Moderate: <1000 to 100 (<3 to 2)	
		Low: <100 (<2)	
14	Persistence (water, soil, sediment, or air half‐life; degradability)	Half‐life (in d)	USEPA 2013
		Very high: >180 (air: >2)	
		High: 60–180	
		Moderate: <60 to ≥16	
		Low: <16 or pass ready biodegradability test not including the 10‐d window	
		Very low: pass biodegradability test with 10‐d window	
15	Environmental fate (water, soil, sediment, or air)	No known classification system; use this to aid in understanding environmental fate	N/A
16	Sewage treatment plant removal	No known classification system; the higher the better	N/A

ACGIH = American Conference of Governmental Industrial Hygienists; ART = Advanced REACH Tool Project; 2D = 2‐dimensional; 3D = 3‐dimensional; BAF = bioaccumulation factor; BCF = bioconcentration factor; MW = molecular weight; N/A = not available; OECD = Organisation for Economic Co‐operation and Development; SMILES = simplified molecular‐input line‐entry system; USEPA = US Environmental Protection Agency; WWTP = waste water treatment plant.

The development of alternatives can go beyond “drop‐in” replacements to also employ alternative product designs as well as ingredients. Product design can play an important role in the potential for exposure to its ingredients and must also be assessed for any changes. Thus, in addition to the properties and parameters that describe the partitioning, transport, fate, and potential for exposure of the substances, we also included a description of the product and a set of 13 parameters (15 including subparameters) related to the function of the ingredient in the product, the product itself, and the use of the product (Table 2). For example, parameters related to the function of the ingredient in the product include a description of the function, the ingredient concentration in product, and the accessibility of the ingredient during product use. Parameters related to the product include form (e.g., powder, liquid, gel), delivery type (e.g., spray, pourable), and disposal method. Finally, parameters related to use patterns include target population and use rate (frequency, duration, and amount). Each of these parameters can be influenced by product design, which could change the potential for exposure.

**Table 2:** Product parameters and classification

Nr	Product parameter	Classification	Source of classification
1	Ingredient function in product	N/A	
2	Life cycle stage	N/A	
3	Exposed populations	N/A	
4	Product form	Formulation: gas > powder > liquid > gel > paste > solid	N/A
		Article: surface coating > homogeneous > encased	
5	Product delivery type	Aerosol > spray > pourable > squeezable	N/A
6	Expected exposure route and/or use pattern	Oral > inhalation > dermal	N/A
7	Frequency, duration, and amount of use	Hourly > daily > weekly > yearly	Orders of magnitude
		Seconds > minutes > hours > days	
		μg > mg > g >	
8	Ingredient concentration in product	Concentration (%)	Orders of magnitude
		>50–100	
		>25–50	
		>10–25	
		>1–10	
		0.1–1	
		<0.1	
9	Ingredient total use volume	Use (in t/y)	Orders of magnitude
		>100 000	
		>10 000–100 000	
		>1000–10 000	
		>100–1000	
		>10–100	
		1–10	
10	Other ingredients in the formula that may differentially impact potential for and type of exposure to the target ingredient and alternative		
11	Accessibility of ingredient in product and during use	Yes or no	N/A
12	Separation potential during product life	Diffusivity or molecular weight	USEPA 2015
13	Product disposal method	Air > down the drain > landfill	N/A

N/A = not available; USEPA = US Environmental Protection Agency.

The comparison of parameters that are important for characterizing exposure, focusing on factors that either are intrinsic to the target and alternative ingredients or are fundamental to the design of the product in which the substances are used, is in line with NAS National Research Council recommendations (NRC 2014b).

The potential for persistence (propensity to remain in the environment for long periods), bioaccumulation (accumulation in primary living organisms via the food chain), and long‐range atmospheric transport has been used for decades to classify chemical substances with respect to the likelihood of environmentally mediated exposure (Mackay et al. 2001). Expanding beyond persistence and bioaccumulation, we set out to identify additional properties and parameters that would enable comparative evaluations of exposure to human and ecological receptors. The goal was to produce a system to allow a qualitative comparison for a specific ingredient performing a specific function in a specific product, such that the range of human and environmental exposure potential would be encompassed.

Screening‐level exposure models are becoming increasingly available, such as ConsExpo (Delmaar et al. 2005), stochastic human exposure and dose simulation–high throughput (SHEDS‐HT) (Isaacs et al. 2014), the European Centre for Ecotoxicology and Toxicology of Chemicals Targeted Risk Assessment (TRA) tool version 3.1 (ECETOC 2014), the European Chemicals Agency Chemical Safety Assessment and Reporting (CHESAR) tool (ECHA 2017a), the Risk Assessment Identification and Ranking (RAIDAR) model (Arnot et al. 2010), and the Performance Improvement Framework (PiF) (Fantke, Ernstoff et al. 2016). Therefore, it is recognized that acquiring, installing, and running such models is often formidable. Moreover, the models at times lack transparency with respect to underlying assumptions, parameter requirements, and processes for determining exposure. Instead, the intention of the present study was to assemble a set of parameters together with generally accepted classification or benchmark values, indicative of either increased or decreased exposure potential that could be evaluated as a preliminary assessment prior to running even simple quantitative exposure models.

To support consistent application of the qualitative methodology, a template (see Document S2, Figure S1) was developed, indicating the ingredient and product parameter information to be collected together with the source reference for the information and identification of data gaps. The other columns of the template allow for documentation of the comparisons, including the anticipated influence on exposure potential, rationale for the influence on exposure potential, relevance and confidence of the collected information for exposure, as well as description of the overall assessment, which is described in more detail in the subsequent sections.

### Classification of exposure parameter information

Establishing criteria for comparing the exposure parameter data is critical to creating a systematic qualitative exposure assessment process for both ingredient‐ and product‐level exposure parameters. This would support a comparison of the exposure potential of ingredients being assessed in different product applications.

Once data are collected, the next step is to compare the target and alternative ingredients on a parameter‐by‐parameter basis. The objective is to compare each parameter to determine whether there is a difference in the behavior of each substance that may contribute to differential emissions and exposure according to predefined classification schemes or criteria (e.g., very soluble versus slightly soluble) as per the USEPA (2013) Sustainable Futures. Identifying the comparisons in which there are classification differences will support an assessment of whether exposure is likely to be lower, higher, or about the same for the alternative ingredient versus the target for different human populations and ecological receptors. Thus, there needs to be a way to judge whether differences between parameters are substantial or not within the definitions of each classification scheme. A useful model is the Globally Harmonized System for Classification and Labeling (GHS), whereby hazard levels are classified into categories (UN 2015). Utilizing these models makes hazard comparisons easier and is a part of many AA hazard assessment frameworks (Jacobs et al. 2016). A similar classification scheme for exposure information did not previously exist.

We investigated sources for a scientific basis or authoritative precedent that could be used to identify such differences for the exposure parameters and summarized the classification approach in Tables 1 and 2. Many parameters were found to have scientific precedents for a numerical scheme that allows for rating or ranking results with respect to the magnitude of the differences to exposure potential. Some parameters can be measured but have no established schemes to judge any differences with respect to their influence on levels of exposure. Other parameters are descriptive and intended to provide context on how a product is designed and used, such as delivery type and product form.

The qualitative methodology template described earlier (Document S2, Figure S1) includes a column for documenting the parameter comparisons as a rating of the exposure impact of any substantial differences: −1 is used to signify that potential exposure from an alternative ingredient is likely to be lower than the target ingredient based on that parameter, 0 if likely to be about the same, and +1 if likely to be higher.

This classification framework enables making judgments about differences in exposures in a standardized way and was applied in the case studies. With ongoing use of this framework, the strengths and weaknesses of the classification scheme can be better understood and the approach for evaluating exposure impact can be revised as needed.

### Relevance, confidence, and data gaps

In developing a qualitative exposure methodology that seeks to determine the potential influence of different parameters on exposure by comparing the values for each parameter, the relevance, confidence in the data, and data gaps should be evaluated and considered, which are summarized in Table 3 and described in the following paragraphs.

**Table 3:** Description of criteria to evaluate relevance, confidence, and data gaps

	Relevance	Confidence	Data gaps
High	All parameters that are associated with the expected primary exposure routes from product use and disposal	The data available for the parameter on both ingredients being compared are measured or derived from measurements and are of good quality.	A “show stopper” because the parameter is associated with the primary exposure route from product use. The overall assessment must clearly reflect a high level of uncertainty.
Medium	All parameters that are associated with the expected secondary exposure route from product use and disposal	The data available for the parameter are of lower or different quality (e.g., estimated on both ingredients; measured on 1 ingredient, estimated on the other; or Klimisch scores are different).	A data gap here introduces some uncertainty, given that the parameter is associated with a secondary exposure route from product use. The overall assessment should offer a caution and indicate the data needed to make a more confident decision.
Low	Any parameter that is not likely to be associated with a relevant expected exposure route from product use and disposal	There are no data available for the parameters on 1 or both ingredients being compared.	A data gap here is not considered relevant, given that the parameter is not likely to be associated with an exposure route from product use. The overall assessment can be made without the need for this information.

For relevance, the methodology addresses the extent to which each parameter is associated with the exposure to the ingredient through its use in a specific product application. The generic conceptual maps for human populations and ecological receptors (Figures 2 and 3) are preliminary tools for identifying the relevance of the parameters. The relevance of a specific parameter is assessed as high, medium, or low, based on the degree of its association with exposure in that product. In cases in which there is a substantial difference indicating an exposure impact in a high‐relevance parameter, this is a strong signal about an important difference in potential exposure between the target and the alternative. A similar situation for a medium‐relevance parameter suggests a less strong, but still present difference.

For confidence, the methodology addresses the degree to which there is an assurance in the data that are being compared, and data confidence is judged to be high, medium, or low. A confidence assessment helps to adjust final determinations in the overall analysis. If a substantial influence on exposure has been identified in a high‐relevance parameter and there is high confidence in the data, this would further strengthen an already strong signal about exposure. An influence on exposure potential with medium relevance would be weakened by medium confidence in the data. It is acknowledged that measurements can vary for several parameters. This issue is more relevant in quantitative AA, where such differences may influence the comparison of alternatives, whereas in a qualitative context, such possible variability in measurement data might be indicated but will usually not influence AA results.

Another aspect of confidence is the selection of data for assessment when there are multiple sources. Different types of input data are required for assessing the potential for exposure in an AA context. To select a specific value from multiple choices, a standard approach and priority for the selection of data to be used in the analysis is needed based on the quality and reliability of the information.

Several large data collection and reporting programs have developed and refined such standard approaches, including the USEPA (1999) and OECD (2009) high production volume (HPV) chemical programs and the Registration, Evaluation, Authorisation and Restriction of Chemicals (REACH; ECHA 2018) program. Alternative assessments such as those on printed circuit boards conducted by the USEPA (2015) Design for the Environment program follow these same standard approaches. These programs have applied a standard hierarchy for the selection of data: experimental preferred over estimated (e.g., from quantitative structure–activity relationships [QSARs]), which is preferred over analog and chemical category approaches. Although some parameters may be measured, this is not feasible for all, in which case estimation is used (e.g., the molecular weight for substances of unknown or variable composition [UVCBs]). High‐throughput data (e.g., the USEPA's Chemistry Dashboard; Williams et al. 2017) and models (e.g., Huang et al. 2016) are becoming more and more available to predict physicochemical properties when measured data are missing.

These data collection programs also consider the reliability of information. Klimisch scores of 1, 2, 3, or 4 indicate classes of data “reliability” obtained from reported compliance with testing guidelines or other standards (e.g., good laboratory practice [GLP]) and the degree to which experimental details are documented as defined in Klimisch et al. (1997). Only Klimisch 1 or 2 scored data should be chosen, given that a score of “3” means the data are unreliable and “4” means reliability is unknown. However, Klimisch scores and similar reliability schemes should be used with caution because they might not be suitable for all relevant AA data.

These existing practices were adopted for the present methodology. Assessors researching information need to document the sources, type, reliability, references, and data preferences (including justification) for information on each parameter. Reporting data preferences is important because measured data might not be available or preferable in all cases. As an example, estimated or archetypal data might be preferred whenever spatiotemporally explicit data are either not applicable (e.g., due to unknown location of exposure) or not desirable (e.g., broad range of consumers exposed or using a product across different regions). Fantke et al. (2014) demonstrated this for pesticide dissipation half‐lives in crops relevant for human exposure via crop residues, in which actual measured half‐lives would be difficult to determine for all possible combinations of pesticides, crops including crop growth stage, soils, climate, application scenarios, and so forth. However, measurement guidelines that are usually followed (e.g., OECD Guidelines for the Testing of Chemicals) might require adaptation to maximize the consistent and optimal use of measured data in existing and newly emerging qualitative and quantitative exposure assessments (Fantke, Arnot et al. 2016).

Finally, there may be data gaps, which are important to note and to determine the relative importance of missing information from one or both ingredients. Data gaps are judged as being of high, medium, or low concern. For high‐concern missing data, it may not be possible to complete the assessment until that data gap is filled. Medium‐concern missing data should be flagged in the overall analysis, with an explanation about their importance to the conclusions of the study and possible need for any further consideration. In cases in which multiple alternatives are being considered, data gaps and uncertainty should provide important weighting to replacement decisions.

The qualitative methodology template (Document S2, Figure S1) includes columns for addressing relevance and comparing data confidence. Data gaps should be identified in the target ingredient or potential replacement data columns.

### Approach to data analysis and overall assessment

Once all the fields in the assessment template (Document S2, Figure S1) are populated, the assessor should now make an overall judgment and provide a rationale about how the exposure to potential alternative ingredients is expected to alter with respect to the original target ingredient. The exposure impact rating of −1, 0, or +1 for each parameter, along with the relevance and confidence scores, needs to be evaluated at this stage to make the overall comparison between both ingredients. The parameters with a “0” exposure impact rating can be initially screened out. The next step should be to consider the parameters for which exposure is likely to be higher or lower to develop an overall impression on the likely direction of change for both human and environmental exposure. This must go beyond a straightforward summation of the positive and negative values because the assessor must also take into consideration the relevance of each parameter to that product type. When exposure is likely to be lower or to be about the same with good certainty, use of the alternative can be supported from a safety standpoint if hazards are also the same or lower, although there are many other factors to consider in an AA decision (NRC 2014b). In cases in which exposure considerations seem to be relevant, in which different exposure routes could lead to trade‐offs, or in which there are high uncertainties, further work is necessary such as a higher tier (quantitative) exposure assessment or the development of information to fill data gaps. This finding generated on the basis of an overall assessment of the scores of high‐ and medium‐relevance parameters should be written in the “overall assessment” section in the assessment template, followed by a brief discussion of the key parameters driving this conclusion, along with important uncertainties and data gaps. The form of the overall assessment is flexible and could also be presented as a cover page for the template. As indicated earlier, the assessor should weigh the confidence rating of all of the parameters while making the final decision. Low confidence in a high‐relevance parameter value would leave room for major uncertainty in the overall assessment and should be reflected as such in the assessor's final recommendation. It is also important to further evaluate the impact scores for medium‐ and low‐relevance parameters to capture the overall range of the exposure variability in the assessment. In cases when low‐relevance parameter scores contradict the finding of the high‐ and medium‐relevance parameters, a cautionary statement pointing out those parameters and how they may impact the overall assessment should also be included in the final recommendation by the assessor.

It is important to note that differences in a single physical or chemical parameter may not necessarily indicate substantial differences in overall exposure. For example, in a scenario in which dermal application has been identified as primary exposure pathway, log *K*_p_ may be used to identify differences in skin penetration. If all other parameters are equal, that may be a sound basis for a decision. However, this is often not the case and if the ingredient with higher potential for skin penetration is also much more volatile, then the volatility may lead to evaporation (and subsequent inhalation) before there is significant skin penetration. Hence, inhalation is another potential exposure route, which requires thorough consideration of volatility‐associated parameters such as vapor pressure, log *K*_oa_, and Henry's Law constant, together with skin absorption parameters. Consequently, the assessor must make an overall judgment considering all parameter comparisons where there are substantial differences. An example of such scenario is presented in the eau de toilette case study in the following section.

Although there are multiple sources of information on certain physicochemical properties of various common compounds, major data gaps exist for new and UVCB compounds (Grimm et al. 2016). Furthermore, the lack of information on certain less studied or difficult to obtain parameters, such as particle attribute size, partitioning coefficients, and ingredient concentration in the final product (Dionisio et al. 2015), makes the AA more challenging. It is recommended that the assessor look at the totality of the information to make the overall judgment and to identify the critical areas of further research to strengthen the interpretation of such analysis. The key data needs that will help increase the confidence in the overall assessment should be specifically listed in the final recommendation by the assessor. Expert review should also be considered to improve consistency and reliability of assessments.

Regardless of the uncertainties associated in the analysis, our qualitative comparison matrix presented here provides a structured framework for incorporating exposure information into AA and can aid in selecting key areas of concerns for conducting higher tier, more detailed, quantitative exposure assessments when necessary. Without quantitative assessment, identification of the most important exposures is difficult. As such, this methodology is beneficial in identifying the general direction of change of likely exposure, but not the amount of change. Therefore, a conservative bias must be applied in overall assessments. To the extent that there is uncertainty or a mix of both higher and lower exposure signals, a higher tier quantitative exposure approach may need to be recommended after considering the general conclusions of the hazard portion of the alternative assessment. It is recognized that although this framework stresses the importance of exposure in evaluating chemical alternatives, it should not be utilized in isolation and must be combined with other approaches and indicators to evaluate toxicity, life cycle impacts, and other factors when considering a higher tier evaluation.

## CASE STUDIES

### Selection from existing AAs

An important aim of the present project was to conduct case studies to test and improve the methodology developed by the group. Existing AA examples using publicly available North American and European sources were consulted, and more than 150 candidates were identified following reviews of AAs published by the USEPA (2016) Design for the Environment program, the Toxics Use Reduction Institute (MA TURI 2013), and the NAS (NRC 2014b) as well as those catalogued in the Alternative Assessment Toolbox (OECD 2016) and the Substitution Support Portal (SUBSPORT 2017). A more detailed description of the case study selection and criteria applied is provided in Supplemental Data Document S1).

The focus for the present project was on developing exposure‐related information according to the methodology for the purpose of identifying whether exposure to the alternative is likely to be higher, lower, or about the same as to the target ingredient. There was no intent to investigate the quality or reliability of the existing hazard assessments, nor was there any endorsement of the findings of those publications.

#### Netherlands National Institute for Public Health and the Environment (RIVM): Eau de toilette – musk xylene

The first case study focused on replacement of musk xylene (CASRN 81‐15‐2) with Muscone (3‐methyl‐cyclopentadecanone) (CASRN 541‐91‐3) in eau de toilette (see Supplemental Data Document S2 for additional details). Conceptual maps for both human populations and ecological receptors were developed (Document S2, Figures S2 and S3, respectively) based on the intended use of the eau de toilette as a perfume. The most relevant exposure pathways for human populations and their pets are

- dermal contact of the product ingredients by the user from application until it is washed off (primary exposure pathway);
- inhalation of volatilized, aerosolized, and/or evaporated product ingredients, either by the user or bystanders (including pets) during application and during the course of the day when the fragrance is on the skin (secondary exposure pathway); and
- inhalation, incidental ingestion, and incidental dermal contact by users and bystanders (including pets) with household dusts that indirectly contain product ingredients (tertiary exposure pathways).

Eventually, the product user will wash the product from the skin, with the remaining product ingredients going down the drain either to surface water, a wastewater treatment plant (WWTP), or groundwater. During the application and the course of the day when the product is on the skin, product ingredients may also evaporate into the indoor or ambient air. The ecological exposure pathways deemed relevant to the subsequent evaluation included exposure routes and ecological receptors associated with several potential exposure media, including WWTP sludge, surface water, sediment, and groundwater (Leonards and de Boer 2004). The rationale for inclusion of these pathways as relevant to the use was based on the conclusion by the PBT Expert Working Group of the Technical Committee of New and Existing Chemicals that musk xylene is a very persistent and very bioaccumulative substance (ECHA 2017b). All of the remaining exposure media and associated exposure routes and receptors were not considered relevant for further evaluation because of the dilution the product ingredients would have when reaching those media (ambient air) or before reaching those media (upland soil, wetland or riparian soil, and diet).

The “target ingredient,” potential replacement,” and associated reference columns in Supplemental Data Document S2, Table S1 list the data that were acquired during the parameter search as well as the classification category assigned to each individual data point. With these assigned classifications, a decision was made for each parameter, by judging whether the exposure for the potential replacement ingredient was likely to be higher, lower, or equal (+1, −1, or 0, respectively) to that of the target ingredient. The rationale for this exposure impact decision was also included. In addition, the evaluator provided a high, medium, or low ranking in regard to the relevance of each parameter to the assessment and in regard to the confidence in the parameter data.

Several of the ingredient and product parameters were considered highly relevant to the qualitative evaluation because of differences between the target compound and the alternative. These parameters include vapor pressure; skin permeability; Henry's Law constant; persistence; half‐lives in water, soil, and sediment; sewage treatment plant removal; and ingredient concentration in product. Other parameters were also assigned a high relevance because of their association with the dermal exposure route or disposal; however, no substantial differences were found between the target compound and the considered alternative according to the classification scheme. Parameters without substantial differences were not considered either individually or in the overall qualitative assessment.

The vapor pressure and Henry's Law constant for Muscone indicate that it will volatilize to air more readily than will musk xylene, shifting the expected route of exposure from dermal to inhalation exposure. The inhalation exposure route, however, was deemed secondary to the dermal exposure route because eau de toilette is a leave‐on personal care product with a short‐lived inhalation exposure potential, primarily only when the fragrance is applied to the skin. Consequently, Muscone was assigned a lower influence on human exposure than was musk xylene for these 2 parameters. On the other hand, the skin permeability for Muscone is >3 orders of magnitude higher than for musk xylene. This parameter, if assessed by itself without consideration of Muscone's volatility, would indicate that Muscone penetrates the skin more readily than does musk xylene. Because the qualitative evaluation of the individual parameters is intended to ensure a conservative outcome, Muscone was therefore assigned a higher influence on exposure for the skin permeability parameter. The confidence assigned to these 3 parameters (vapor pressure, Henry's Law constant, and skin permeability) was a “medium” designation because in every case the data points were estimated. Muscone was also assigned a higher exposure impact than musk xylene for ingredient concentration in the product, although the confidence in this parameter is low because the concentration for Muscone in eau de toilette is essentially not publicly available information. It was estimated using a generic default (5%) from the RIVM ConsExpo consumer exposure model (RIVM 2016). On the other hand, the European Cosmetics Directive limits musk xylene to 0.4% (EC 2004).

Numerous ingredient parameters indicative of persistence in the environment, including water, soil, sediment, and air half‐lives, suggested a lower potential for exposure for Muscone when compared to musk xylene. The sewage treatment removal parameter likewise indicated a lower exposure potential for Muscone than for musk xylene, given that the removal percentage was estimated to be nearly twice that of musk xylene. A “high” category was assigned to the relevance for all of these parameters because of conclusions drawn regarding the persistence and bioaccumulative nature of musk xylene (ECHA 2017b). A “medium” category was assigned to the confidence for these parameters because they were estimated.

Based on the ingredient parameter and product ingredient qualitative evaluation, human exposure to the potential alternative (Muscone) is likely to be about the same, given the 4 most highly relevant parameters offset each other (2 indicate that the exposure potential for Muscone is lower than for musk xylene, and two indicate that the exposure potential for Muscone is higher than for musk xylene). In addition, there is an indication that the inhalation pathway would be the most relevant for Muscone, whereas the most relevant pathway for musk xylene would be dermal contact, which was deemed the primary exposure pathway based on the use of the eau de toilette. Presumably, product users would have much shorter exposure durations for inhalation of the product when compared to dermal contact with the product. For the environment, exposure to Muscone is also likely to be about the same as musk xylene because the most relevant parameters offset each other. Muscone may have a lower persistence in the environment because of shorter half‐life values and a higher sewage treatment removal rate, but the potentially increased concentrations eventually reaching the environment may act to counterbalance this presumption.

The key uncertainties associated with this evaluation include the following:

- Maximum concentration of Muscone in the fragrance
- Significance of the shift of the predominant exposure route from dermal contact (musk xylene) to inhalation (Muscone). Comparison of the inhalation and dermal exposure benchmarks for the 2 substances is needed to determine whether the presumed shorter exposure duration for the inhalation pathway equates to a lower risk potential. In addition, an assessment of the competition between dermal exposure and volatilization is needed, with a high volatilization having the potential to substantially reduce dermal exposure. This is especially relevant for this leave‐on cosmetic case study, for which the higher skin permeation of Muscone may be at least partly compensated by its higher volatilization compared to musk xylene. This would require the use of quantitative approaches that account for these competing exposure and removal pathways (Csiszar et al. 2016, 2017; Ernstoff et al. 2016).

For this evaluation, 1 parameter had an exposure‐related data gap: particle attribute size because of a lack of data. This parameter is associated with the secondary exposure route (inhalation), thus indicating a “medium concern.”

Due to the offsetting conclusions regarding the key parameters and the uncertainties determined by this qualitative evaluation, the overall recommendation is to advance to a higher tier assessment in which exposure is quantified, after considering any differences between the target and proposed alternative in the hazard assessment aspect of the AA.

#### Danish Environmental Protection Agency: Toys – DEHP

The second case study addressed the proposed replacement of di(2‐ethylhexyl) phthalate (CASRN 117‐81‐7), more commonly known as DEHP, with its structural isomer di(2‐ethylhexyl) terephthalate (CASRN 6422‐86‐2), or DEHT, in toys (see Supplemental Information Document S2 for additional details). Although these 2 compounds have the same molecular formula, the substituent chains on DEHP are in the *ortho* position, making it a phthalic ester, whereas the chains of DEHT are in the *para* position, making it a terephthalic ester. The distinction is important because phthalic esters (commonly referred to as “phthalates”) have been associated with reproductive toxicity; it appears that the *para* position allows complete metabolism to take place, but the *ortho* position does not (Wirnitzer et al. 2011).

Conceptual maps for exposure of both human populations and ecological receptors were developed (Document S2, Figures S4 and S5, respectively) based on the specific use of the substance, namely as a plasticizer in toys. *Ortho*‐phthalate esters (particularly DEHP) are used as plasticizers to impart flexibility to polyvinyl chloride (PVC). Polyvinyl chloride is the world's 3^rd^‐most widely produced synthetic plastic polymer (Allsopp and Vianello 2012) and is widely used in consumer products such as children's toys, cosmetics, medical devices, flooring, water piping, and food packaging (Xie et al. 2016). Because phthalates are not chemically bound to PVC, they can easily migrate toward whatever is in contact with the surface of the toy, potentially exposing the user through inhalation, ingestion, and dermal absorption (Little et al. 2012). Indeed, metabolites are found ubiquitously in the urine of humans, often with significantly higher levels among children than adults (Becker et al. 2009). The focus of the present case study was on the intended use in toys—objects designed to be played with, generally by children and pets. Children may have frequent contact with toys for long periods each day; moreover, children have been observed to mouth toys more frequently than even their hands (Tulve et al. 2002). The most relevant primary exposure pathways for human populations were determined to be via ingestion and dermal contact.

The ecological exposure scenario assumed was disposal in landfills and incineration. As such, the only pathways deemed relevant were those that involved fate after landfill disposal, namely, migration through soil into groundwater and bioaccumulation in the food chain. Additionally, persistence was also considered relevant.

On the basis of these highlighted exposure pathways, the most relevant exposure parameters were considered to be solubility in water, predicted percent human oral absorption due to mouthing behavior in children, skin permeability, log *K*_ow_, BCF, and soil sorption coefficient. All “product exposure parameters” were deemed highly relevant. However, only 1 difference existed between the target ingredient and replacement ingredient (i.e., concentration in product), and it was negligible.

The overall assessment is that potential exposure to the alternative is likely to be about the same or with the possibility of being slightly lower (Document S2, Table S2). The potential replacement has similar properties to the target, but for the specific application in children's toys, the replacement would be slightly preferred due to lower water solubility, which may result in less migration to saliva during mouthing by children and subsequently lower intake. The potential replacement would also be preferred due to lower skin permeability. These advantages, however, are tempered by the higher log *K*_ow_, which suggests easier absorption and longer half‐life in the body. Because there are competing parameters of high relevance in the qualitative assessment, a higher tier, quantitative assessment of the primary exposure routes may be appropriate, after considering any differences between the target and proposed alternative in the hazard assessment aspect of the AA. There were no meaningful differences in parameters related to the environment; thus, environmental exposure is assumed likely to be about the same. The data gaps that emerged through this analysis included the following: particle attribute size (low relevance) and separation potential during product life (medium relevance). The key uncertainties or data needs involve the rate of migration to the surface of the toy for each substance. That information would allow a better assessment of transfer from the object into the child's saliva during mouthing and onto the child's skin during other contact. In addition, a quantitative assessment would address uncertainties in the magnitude of competing high‐relevance parameters.

## CONCLUSIONS AND FUTURE RESEARCH NEEDS

The objective of the present paper was to develop a methodology for integrating qualitative exposure information into the existing AA process. A methodology to accomplish this for single‐ingredient replacement was developed, and 2 case studies were conducted that helped to evaluate and improve the methodology in an iterative fashion. The methodology and case studies presented herein represent the finalized approach. Additional case studies might indicate other scenarios and product applications for which differences in exposure might be even more relevant, and these should be further explored (e.g., Fantke, Ernstoff et al. 2016).

This concept is easy to understand, interpret, and communicate. It is a stepwise protocol and procedure that considers all key components of potential human and environmental exposure and is a useful way to structure expert knowledge in a qualitative way. Finally, it provides a basis for making a judgment as to whether exposure is likely to be about the same or lower for any considered alternative ingredient or whether exposure is likely to be higher or different in route, which would indicate the need for higher tier and more quantitative exposure assessments. As noted in the case studies, when exposures are likely to be about the same or even lower but there are significant uncertainties, the need for higher tier and more quantitative assessments may be necessary. The results of the comparative hazard assessment should also be considered before conducting higher tier assessments.

Most importantly, the present work has demonstrated that different ingredients that are potential alternatives for a target substance in the same product application can yield different overall human and/or ecological exposure. Consequently, adding exposure information is essential and can improve overall AA decisions, minimizing regrettable substitution, which could occur when simply assuming equal exposure to replacement ingredients without having evaluated exposure either qualitatively or quantitatively. Collecting and assessing exposure information requires exposure expertise, which has not typically been a part of most existing AA efforts. Similar to the hazard aspect of AAs, there will likely be data gaps for key information, which, if not addressed, can create uncertainty in any assessment conclusions.

Adding the collection and assessment of several dozen additional parameters to an AA will clearly increase the effort required for conducting an AA. With a systematized and clarified protocol described herein and with a learning curve from doing multiple assessments, effort should be reduced to a reasonable level in the context of improving AA decision making. By applying the presented qualitative exposure assessment framework to further cases, more can be learned about the strengths and weaknesses of the methodology, and the methodology can be revised as needed.

Regarding future research needs, the time available and scope of the present project did not allow for addressing several topics that arose. No existing AA that addressed fundamental product design change was found to be adequate for a case study; thus, that aspect of the methodology has not been fully evaluated. Multiple ingredient replacements and/or concentration changes are typical in real‐world reformulations to improve product safety (Thomas 2014), and those situations are not able to be addressed with the present methodology. Finally, the focus of the present effort was to explore a qualitative comparative approach. The presented qualitative methodology can usually identify the general direction of change of likely exposures. However, pinpointing the most important exposures and their magnitude may be a challenge; thus, a conservative bias should be applied in overall assessments. In cases where a qualitative approach to AA (addressing both exposure and hazard) is insufficient to distinguish substantial differences between original chemicals in products and their potential alternatives, quantitative approaches might be explored to refine initial results. Quantitative approaches, however, also come with additional data and modeling requirements that would need to be addressed. Initial quantitative frameworks for exposure assessment are already available for application in AA (NRC 2014a; Fantke, Ernstoff et al. 2016), but these need to be extended to different product exposure scenarios and respective exposure pathways that might become relevant as a function of assessed product exposure scenario. Huang et al. (2016) provide an overview of a wide range of potentially relevant pathways and modeling approaches for characterizing these pathways in an AA context.

In summary, we agree with NAS that adding exposure information can help to improve AA decision making, and it can and should be a part of every AA. A comparative qualitative approach can serve as an effective initial tier of exposure assessment in the AA context.

## Disclaimer

The views expressed in this article are those of the authors and do not necessarily represent the views or policies of the US Environmental Protection Agency.

## SUPPLEMENTAL DATA

**Document S1.** Case study selection criteria

**Document S2.** Additional supporting figures and tables

**Figure S1.** Template for documenting the qualitative exposure assessment.

**Figure S2.** Conceptual map for human populations from eau de toilette use.

**Figure S3.** Conceptual map for ecological receptors from eau de toilette use.

**Figure S4.** Conceptual map for human populations from toy use.

**Figure S5.** Conceptual map for ecological receptors from toy use.

**Table S1.** Data and evaluation for eau de toilette case study

**Table S2.** Data and evaluation for toy case study

## Supporting information

This article contains online‐only Supplemental Data.

Supporting Information S1.

Supporting Information S2.

## Data Availability

Data and associated metadata and calculation tools for this research will be available upon request from the corresponding author, Michelle Embry, at membry@hesiglobal.org.
