# Supplementary material for: Qualitative Approach to Comparative Exposure in Alternatives Assessment
Source: Integr Environ Assess Manag. 2018 Jul 19;15(6):880–94. doi: 10.1002/ieam.4070 (PMC6899567; doi:10.1002/ieam.4070)
Supplement: Supplementary file 1 — Supporting Information S1. [file IEAM-15-880-s001.docx]

**SUPPLEMENTAL INFORMATION S1**

**Description of case study selection criteria**

Case Studies were selected from examples that focused on the use and disposal of specific products. Applying that criterion, the field of AA examples was narrowed to about 3 dozen promising candidates. The top candidates were selected for the project’s case studies by applying the following additional criteria:

- Products must be discrete formulations or articles.
- The alternative(s) must be single (drop-in) ingredient replacements. Addressing mixture changes was beyond the scope of the project.
- The existing AA must go beyond general high-level “success stories” to include a discussion of the hazard assessment and other considerations.
- The existing AA must include key hazard information that could help point to the type of exposure information that might be most helpful.
- The existing AA must address specific ingredients with Chemical Abstracts Service registration numbers (CASRNs); trade names, proprietary substances, and mixtures would make it challenging to develop physical/chemical property information.
- To better test the exposure methodology, the selected examples should provide a mix of different products, substances, functional uses, and exposure routes.

Some existing examples included multiple alternatives for the target ingredient. For these case studies, just 1 alternative was assessed for illustration purposes. The existing product AAs that were selected for case study were on eau de toilette conducted by the Netherlands National Institute for Public Health and the Environment (RIVM) (2009) and on toys conducted by the Danish EPA (Maag et al. 2010).

**REFERENCES**

Maag J, Lassen C, Brandt UK, Kjoholt J, Molander L, Mikkelsen SH. 2010. Identification and assessment of alternatives to selected phthalates. Copenhagen (Denmark): Danish Environmental Protection Agency. Report no. 1341 2010.

[RIVM] Netherlands National Institute for Public Health and the Environment. 2009. Data on manufacture, import, export, uses and releases of musk xylene. Bilthoven (The Netherlands): RIVM.
