# Supplementary material for: Qualitative Approach to Comparative Exposure in Alternatives Assessment
Source: Integr Environ Assess Manag. 2018 Jul 19;15(6):880–94. doi: 10.1002/ieam.4070 (PMC6899567; doi:10.1002/ieam.4070)
Supplement: Supplementary file 2 — Supporting Information S2. [file IEAM-15-880-s002.docx]

**SUPPLEMENTAL INFORMATION DOCUMENT S2**

**Figure S1.** Template for documenting the qualitative exposure assessment.

**
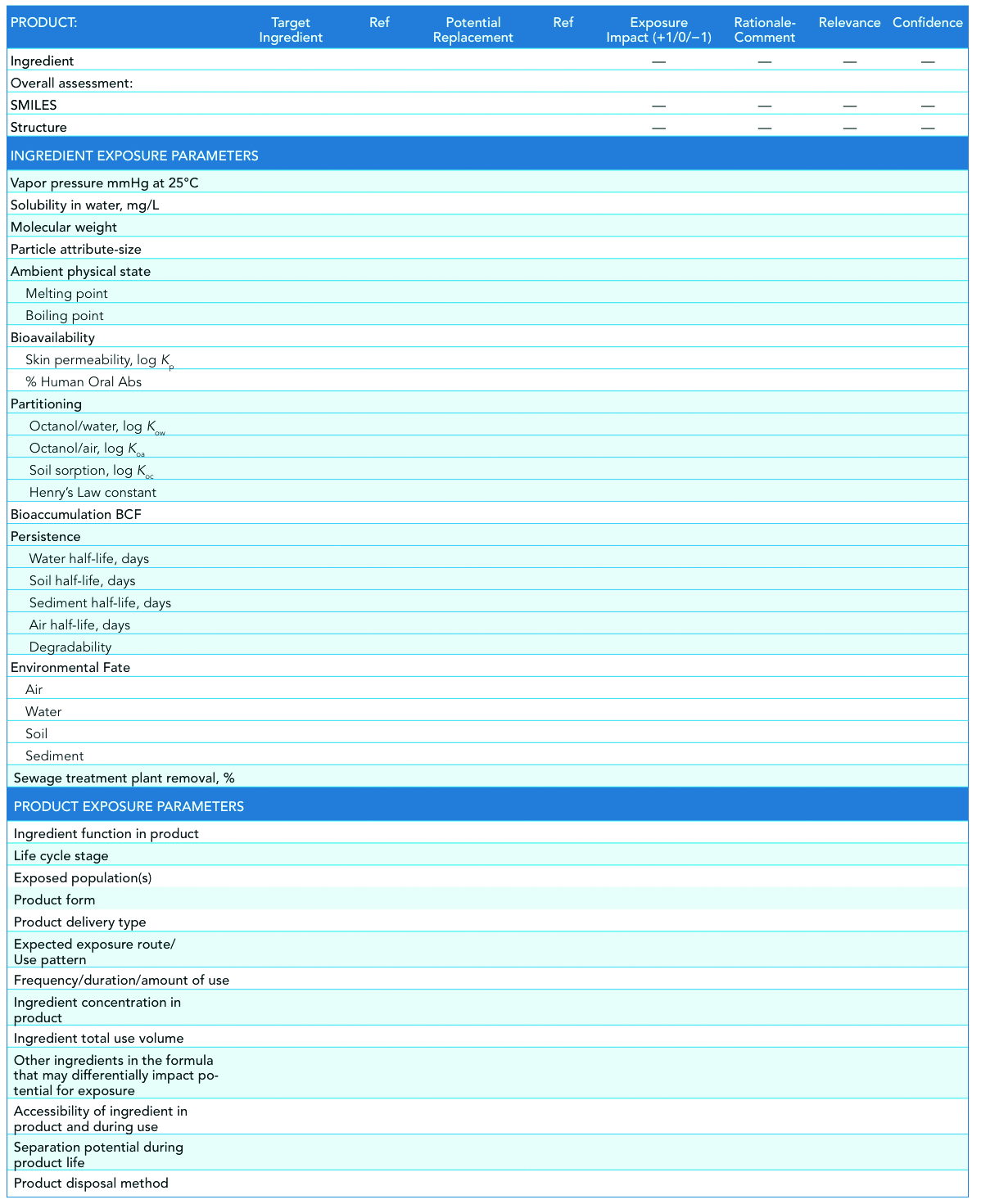
**

**Supporting information for case studies**

**Figure S2. Conceptual map for human populations from eau de toilette use**

**
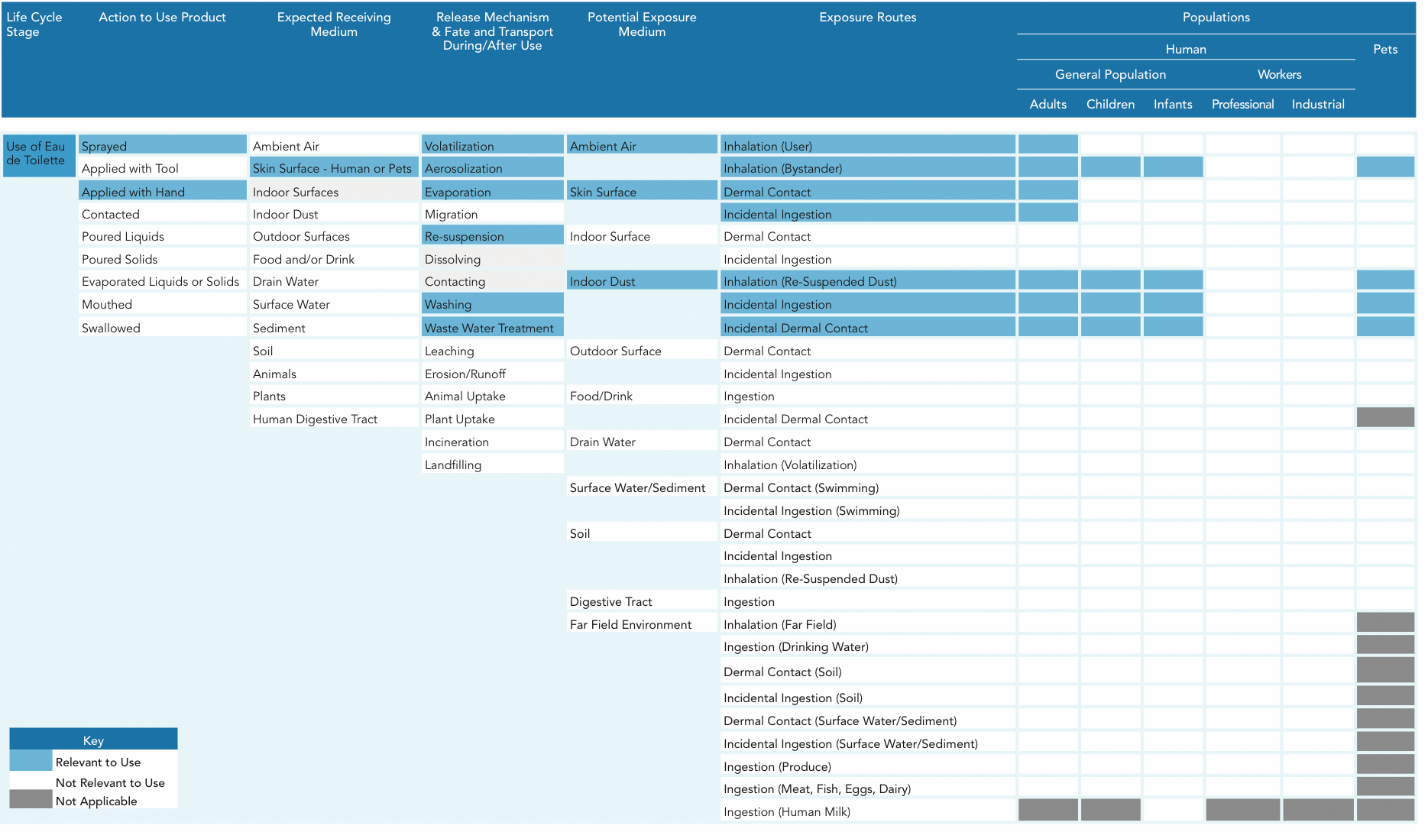
**

**Figure S3. Conceptual map for ecological receptors from eau de toilette use**


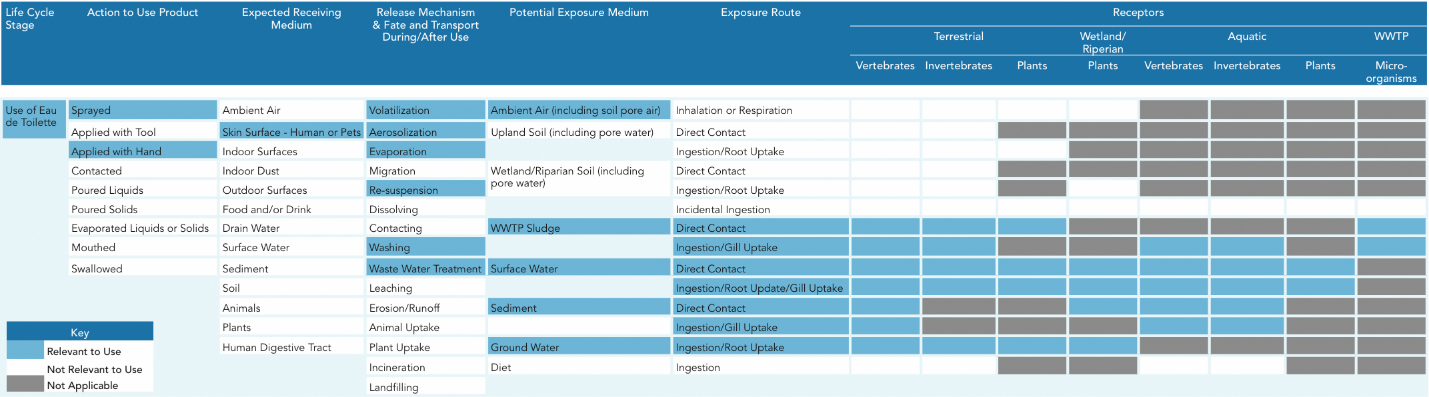


**Table S1. Data and evaluation for eau de toilette case study**

| **Eau de toilette 0.4% max *** | **Target Ingredient** | **Ref** | **Potential Replacement** | **Ref** | **Exposure Impact (+1/0/−1)** | **Rationale-Comment** | **Relevance** | **Confidence** |
| --- | --- | --- | --- | --- | --- | --- | --- | --- |
| **Ingredient** | Musk xylene (CASRN 81-15-2) |  | 3-methyl-cyclopentadecanone (Muscone) 541-91-3 |  | N/A | N/A | N/A | N/A |
| Overall assessment: Exposure to the alternative is likely to be about the same. The most relevant parameters offset each other for both the human and environmental evaluations. In addition, there is indication that the inhalation pathway would be the most relevant for Muscone, while the most relevant pathway for musk xylene would be dermal. Presumably, product users would have a much shorter exposure duration from the inhalation pathway than for the dermal pathway. | | | | | | | | |
| Key uncertainties/data needs: 1) Understand the maximum concentration of the alternative in the fragrance, which will have a significant impact on the extent of human and environmental exposure. 2) A comparison of inhalation and dermal exposure benchmarks for the 2 substances would help determine if this shorter exposure duration equates to a lower risk potential. 3) Assessing the competition between dermal exposure and volatilization for Muscone. Recommend advancing to a quantitative exposure assessment. | | | | | | | | |
| Data gaps: There is one parameter with an exposure impact data gap: Particle attribute size because of lack of data. This parameter is associated with the secondary exposure route (inhalation), thus indicating a "Medium Concern." | | | | | | | | |
| **SMILES** | Cc1c(c(c(c(c1N(=O)(=O))C(C)(C)C)N(=O)(=O))C)N(=O)(=O) |  | CC1CCCCCCCCCCCCC(=O)C1 |  | N/A | N/A | N/A | N/A |
| **Structure** |  |  |  |  | N/A | N/A | N/A | N/A |
| ***INGREDIENT EXPOSURE PARAMETERS*** | |  |  |  |  |  |  |  |
| **Vapor pressure** | 6.35E-07 mm Hg @ 25°C *Vapor-particulate phase* | USEPA (2012) | 0.000469 mm Hg @ 25°C *Mostly vapor phase* | USEPA (2012) | **−1 (derm) +1 (inh)** | **−**1 for dermal because evaporates faster; + 1 for inh because more volatile | High because parameter indicates in this circumstance the dominant exposure route for each compound | Medium because both estimated data |
| **Solubility in water** | 0.15 mg/L (meas) *Slightly soluble >0.1–100* | ECHA (2005) | 0.2213 mg/L *Slightly soluble >0.1–100* | SRC (2016) | **0** | Even if use higher value of 0.8214 for musk xylene, which is calculated, both have same classification | High for human because associated with dermal pathway; High for environment because fraction washed-off will go to the environment | Medium because mix of measured and estimated data |
|  | 0.8214 mg/L (calc) | USEPA (2012) |  |  |  |  |  |  |
| **Molecular weight** | 297.3 <500 g/mol | ECHA (2005) | 238.41 <500 g/mol | HSDB (TOXNET 2016) | **0** | Both have potential for dermal absorption | High because associated with fraction dermal | Medium because mix of measured and estimated data |
| **Particle attribute size** | 100% v/v < 100 µm | ECHA (2005) |  |  | **?** | Cannot determine because data not available for Muscone and do not know the test method used to determine results for musk xylene or the application it represents | Medium because parameter is associated with inhalation pathway, which is a secondary exposure route | Low because data not available for both substances |
|  | 21.8% v/v < 10 µm |  |  |  |  |  |  |  |
|  | 14.4% v/v < 4 µm |  |  |  |  |  |  |  |
| **Ambient physical state** |  | ECHA (2005), USEPA (2012) |  | Haynes (1998), USEPA (2012) | **0** | Both are solids at ambient temperature when pure substances, but product is liquid form | Low because during use, not in pure substance form | Medium because mix of measured and estimated data |
| Boiling point | 411.56°C |  | 329°C (lit) |  |  |  |  |  |
| Melting point | 112–114°C (meas) *Solid when pure substance* |  | 51.13°C (weighted value) *Solid when pure substance* |  |  |  |  |  |
| **Bioavailability** |  |  |  |  |  |  |  |  |
| Predicted skin permeability, log *K*p | −4.749 | USEPA ACToR (2017) | −1.831 | USEPA ACToR (2017) | **+1** | Will penetrate skin more readily | High because associated with fraction dermal | Medium because both estimated data |
| % Human oral Abs | 77 | USEPA ACToR (2017) | 100 | USEPA ACToR (2017) | **0** | Not likely to be orally ingested | Low because associated with ingestion pathway | Medium because both estimated data |
| Octanol/water, log *K*_ow_ | 4.9 A (meas) *Not very soluble in water* | ECHA (2005) | 5.96 *Not very soluble in water* | USEPA (2012) | **0** | Both are not very soluble in water | High because associated with fraction dermal | Medium because mix of measured and estimated data |
| Octanol/air, log *K*_oa_ | 11.821 | USEPA (2012) | 7.409 | USEPA (2012) | **0** | Both have a strong association with lipid or organic surfaces; both are potentially bioaccumulative in the food chain | High because parameter is associated with bioaccumulative and elimination potential | Medium because both estimated data |
| Soil sorption, log *K*_oc_ | 3.59 | USEPA ACToR (2017) | 3.78 | USEPA ACToR (2017) | **0** | Both have strong sorption | High because fraction washed-off will go to the environment | Medium because both estimated data |
| Henry's Law constant | 1.04E-09 atm m^3^/mol *Nonvolatile from water* | USEPA (2012) | 0.000869 atm m^3^/mol *Moderately volatile from water* | SRC (2016) | **−1** | Inhalation pathway focus for Muscone rather than dermal pathway and the opposite for musk xylene | High because associated with fraction volatilized from liquid | Medium because both estimated data |
| **Bioaccumulation (BCF)** | 4400 L/kg (meas) *High* | ECHA (2005) | 4000 L/kg (calc) *High* | USEPA (2018) | **0** | Both have high potential for bioaccumulation | High because fraction washed-off will go to the environment | Medium because mix of measured and estimated data |
| **Persistence** |  |  |  |  |  |  |  |  |
| Water half-life, d | 180  *High* | USEPA (2018) | 38  *Moderate* | USEPA (2018) | **−1** | Muscone less persistent than musk xylene; sediment exposure impact "**−**1" because less getting to the sediment according to environmental fate predictions | High because fraction washed-off will go to the environment | Medium because both sets are estimated data |
| Soil half-life, d | 360  *Very high* | USEPA (2018) | 75  *High* | USEPA (2018) | **−1** |  |  |  |
| Sediment half-life, d | 1600  *Very high* | USEPA (2018) | 340  *Very high* | USEPA (2018) | **−1** |  |  |  |
| Air half-life, d | 13  *Moderate* | USEPA (2018) | 0.36  *Low* | USEPA (2018) | **−1** |  |  |  |
| Degradability | Not readily biodegradable, not expected to hydrolyze, minimal photodegradation | ECHA (2005) | Does not biograde fast | USEPA (2012) | **0** | Both do not degrade quickly | High because fraction washed-off will go to the environment | Medium because mix of measured and estimated data |
| **Environmental Fate** |  |  |  | USEPA (2012) |  |  | High because fraction washed-off will go to the environment |  |
| Water | 3 | USEPA (2012) | 10 | USEPA (2012) | **0** | Slightly more to water; slightly less to sediment |  |  |
| Soil | 85 | USEPA (2012) | 86 | USEPA (2012) | **0** |  |  | N/A |
| Sediment | 12 | USEPA (2012) | 4 | USEPA (2012) | **0** |  |  |  |
| Air | N/A | USEPA (2012) | 1 | USEPA (2012) | **0** |  |  |  |
| Sewage treatment plant removal, % | 53 | USEPA (2012) | 92 | USEPA (2012) | **−1** | Less likely to end up in drinking water and aquatic organisms | High because fraction washed-off will go to the environment | Medium because both sets are estimated data |
| ***PRODUCT EXPOSURE PARAMETERS*** |  |  |  |  |  |  |  |  |
| **Ingredient function in product** | Fragrance, fixative |  | Fragrance, fixative |  |  |  |  |  |
| **Life cycle stage** | Use/disposal phases |  | Use/disposal phases |  |  |  |  |  |
| **Exposed population(s)** | Adult |  | Adult |  | **0** |  |  |  |
| **Product form** | Liquid formulation |  | Liquid formulation |  | **0** |  |  |  |
| **Product delivery type** | Spray |  | Spray |  | **0** |  |  |  |
| **Expected exposure route/use pattern** | Inhalation, dermal |  | Inhalation, dermal |  | **0** |  |  |  |
| **Frequency/duration/amount of use** | 1095/y; 5 min (inh); 0.08 min spray duration (inh); 320 min (derm) | ConsExpo 4.1 (RIVM 2016) | 1095/y; 5 min (inh); 0.08 min spray duration (inh); 320 min (derm) | ConsExpo 4.1 (RIVM 2016) | **0** |  | High because duration on skin relates to fraction washed off | Medium to High (3 out of highest confidence of 4) according to ConsExpo 4.1 for frequency; Low to Medium (2 out of 4) according to ConsExpo 4.1 for duration and dermal amount of use |
|  | Amount:  0.625 m^3^ cloud volume (inh); 0.61 g (derm) |  | Amount:  0.625 m^3^ cloud volume (inh); 0.61 g (derm) |  |  |  |  |  |
| **Ingredient concentration in product** | 0.4% max***  *Per EU Cosmetic law* |  | Up to 5% | ConsExpo 4.1 (RIVM 2016) | **+1** | ConsExpo uses 5% default nonvolatile | High because associated with both dermal and inhalation exposure pathways | Low to Medium (2 out of highest confidence of 4) according to ConsExpo 4.1 for default nonvolatile concentration |
| **Ingredient total use volume** | Base |  | 5.0/0.4 = up to 12.5 times higher |  | **+1** | Proportional to concentration assuming similar annual sales volume | High because fraction washed-off will go to the environment | Low to Medium (2 out of highest confidence of 4) according to ConsExpo 4.1 for default nonvolatile concentration |
| **Other ingredient changes in the formula that may differentially impact potential for exposure** | N/A |  | N/A |  | **0** |  |  |  |
| **Accessibility of ingredient in product and during use** | Accessible in use: mist, on skin |  | Accessible in use: mist, on skin |  | **0** |  |  |  |
| **Separation potential during product life** | N/A |  | N/A |  | **0** |  |  |  |
| **Product disposal method** | Air, water |  | Air, water |  | **0** |  |  |  |

N/A = not available.

**Figure S4. Conceptual map for human populations from toy use**

**
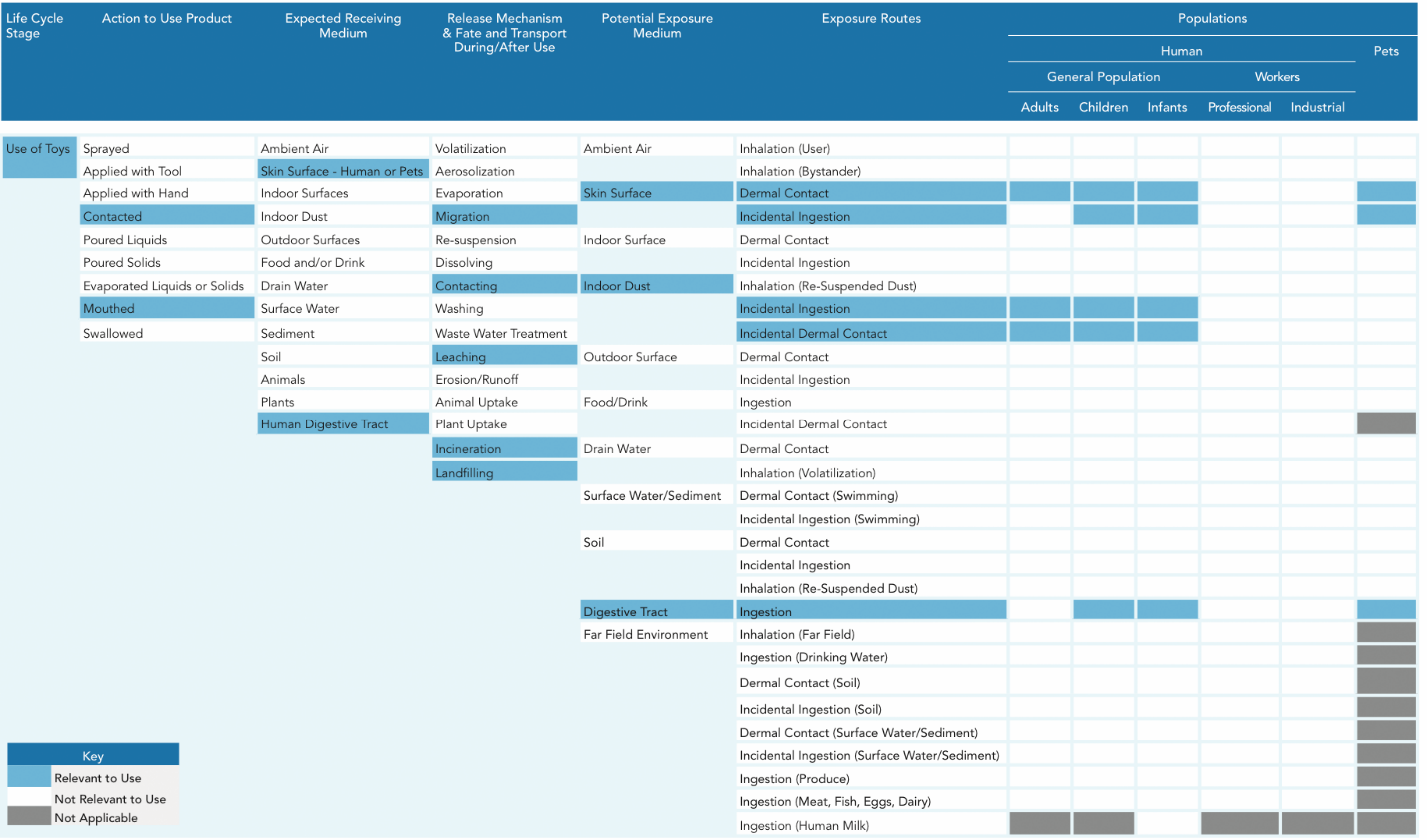
**

**Figure S5. Conceptual map for ecological receptors from toy use**


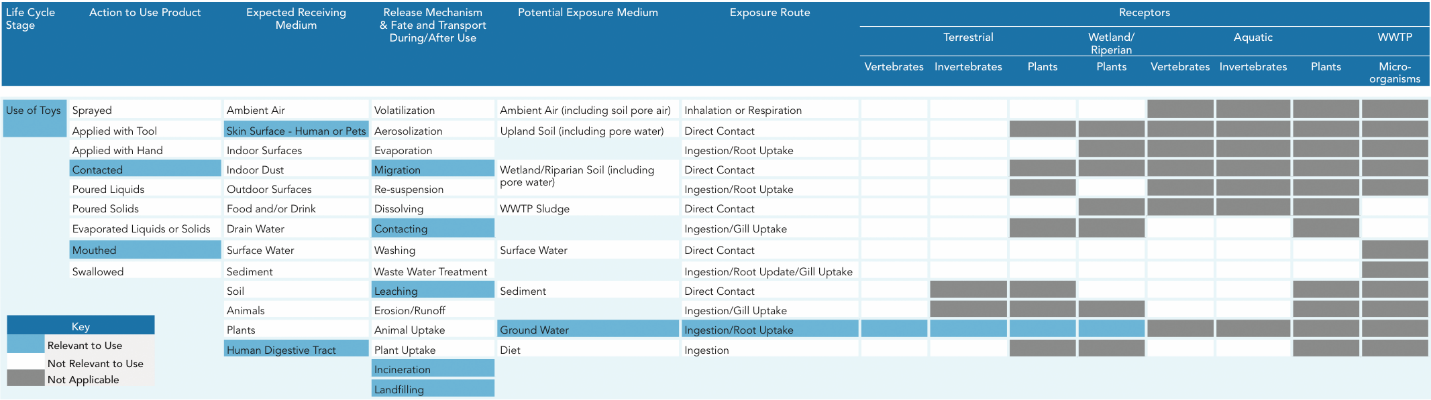


**Table S2. Data and evaluation for toy case study**

| **Toys** | **Target Ingredient** | **Ref** | **Potential Replacement** | **Ref** | **Exposure Impact (+1/0/−1)** | | **Rationale-Comment** | **Relevance** | **Confidence** |
| --- | --- | --- | --- | --- | --- | --- | --- | --- | --- |
| **Ingredient** | di(2-ethylhexyl) phthalate (DEHP) (CASRN 117-81-7) |  | di(2-ethyl-hexyl) terephthalate (DEHT) (CASRN 6422-86-2) |  | N/A | | N/A | N/A | N/A |
| Overall assessment: Exposure to the alternative is likely to be about the same or lower. Potential replacement has similar properties to the target, but for the specific application in children's toys the replacement is slightly preferred due to the effect of lower solubility and children's mouthing behavior. The advantage is tempered by the higher logP, which suggests easier absorption and longer half-life in body. | | | | | | | | | |
| Data gaps: 1) Particle attribute size (Low Impact); 2) Separation potential during product life (Medium Impact). | | | | | | | | | |
| Key uncertainties/data needs: 1) Migration rate for each substance. | | | | | | | | | |
| **SMILES** | CCCCC(CC)COC(=O)c1ccccc1C(=O)OCC(CC)CCCC |  | CCCCC(CC)COC(=O)c1ccc(cc1)C(=O)OCC(CC)CCCC |  | | N/A | N/A | N/A | N/A |
| **Structure** | 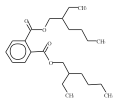 |  | 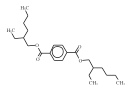 |  | | N/A | N/A | N/A | N/A |
| ***INGREDIENT EXPOSURE PARAMETERS*** |  |  |  |  | |  |  |  |  |
| **Vapor pressure mmHg at 25°C** | 2.03 E-05 *Vapor-particulate phase* | USEPA ACToR (2017) | 2.14E-05  *Vapor-particulate phase* | USEPA ACToR (2017) | | **0** | Negligible difference | Low because trapped in a polymer | Medium because not known if predicted |
| **Solubility in water, mg/L** | 0.001132 *Negligible solubility* | USEPA ACToR (2017) | 0.0002387 *Insoluble* | USEPA ACToR (2017) | | **−1** | Less likely to get into saliva or sweat | High because of children’s mouthing behavior | High |
| **Molecular weight** | 390.56 | USEPA ACToR (2017) | 390.56 | USEPA ACToR (2017) | | **0** |  | High because absorption relevant if <500 g/mol | High |
| **Particle attribute size** | N/A |  | N/A |  | | **?** |  | Low because of product characteristics | Data gap |
| **Ambient physical state** |  | USEPA ACToR (2017) |  | USEPA ACToR (2017) | | **0** | No meaningful difference | Low because of product characteristics | High |
| Melting point | −55 or −50°C |  | −48°C |  |  |  |  |  |  |
| Boiling point | 385°C  *Liquid* |  | 383°C  *Liquid* |  |  |  |  |  |  |
| **Bioavailability** |  |  |  |  | |  |  |  |  |
| Predicted skin permeability, log *K*_p_ | −0.726 | USEPA ACToR (2017) | −1.267 | USEPA ACToR (2017) | | **−1** | Less permeable | High because of expected dermal contact | Medium because predicted |
| % Human oral Abs | 100 | USEPA ACToR (2017) | 100 | USEPA ACToR (2017) | | **0** |  | High because of children’s' mouthing behavior | Medium because predicted |
| Octanol/water, log *K*_ow_ | 7.6 | USEPA ACToR (2017) | 8.39 | USEPA ACToR (2017) | | **+1** | Stays in body longer | High because of many ADME and exposure parameters | Medium because predicted |
| Octanol/air, log *K*_oa_ | 12.56 | USEPA ACToR (2017) | 11.71 | USEPA ACToR (2017) | | **?** |  | Low because of product characteristics | Medium because predicted |
| Soil sorption, log *K*_oc_ | 5.68 | USEPA ACToR (2017) | 5.21 | USEPA ACToR (2017) | | **0** |  |  | Medium because predicted |
| Henry's Law constant (atm m^3^/mol) | 4.37 × 10^−5^ *Moderately volatile from water* | USEPA ACToR (2017) | 1.02 × 10^−5^ *Moderately volatile from water* | USEPA ACToR (2017) | | **0** | No meaningful difference | Low because of product characteristics | Medium because not known if predicted |
| **Bioaccumulation BCF** | 1700  *High* | USEPA (2018) | 700 *Moderate* | USEPA (2018) | | **−1** | less likely to end up in food chain | High because of fate after disposal | Medium because predicted |
| **Persistence** |  | USEPA (2018) |  | USEPA (2018) | |  | No difference in modeled persistence | High because of fate after disposal | Medium because predicted |
| Water half-life, d | 15 | USEPA (2018) | 15 | USEPA (2018) | | **0** |  |  |  |
| Soil half-life, d | 30 | USEPA (2018) | 30 | USEPA (2018) | | **0** |  |  |  |
| Sediment half-life, d | 135 | USEPA (2018) | 135 | USEPA (2018) | | **0** |  |  |  |
| Air half-life, d | 0.49 | USEPA (2018) | 0.49 | USEPA (2018) | | **0** |  |  |  |
| Degradability | Readily degradable | USEPA (2012) | Readily degradable | USEPA (2012) | | **0** |  | High because of fate after disposal | Medium because predicted |
| **Environmental Fate** |  |  |  |  | |  |  |  |  |
| Water | 17 | USEPA ACToR (2017) | 23 | USEPA ACToR (2017) | |  | Slightly more to water and soil; less to sediment |  |  |
| Soil | 63 | USEPA ACToR (2017) | 71 | USEPA ACToR (2017) | |  |  |  |  |
| Sediment | 19 | USEPA ACToR (2017) | 6 | USEPA ACToR (2017) | |  |  |  |  |
| Air | 1 | USEPA ACToR (2017) | 1 | USEPA ACToR (2017) | |  |  |  |  |
| Sewage treatment plant removal, % | 94 | USEPA ACToR (2017) | 94 | USEPA ACToR (2017) | | **0** |  | Low because product not likely to end up in STP | Medium because predicted |
| ***PRODUCT EXPOSURE PARAMETERS*** |  |  |  |  | |  |  |  |  |
| **Ingredient Function in Product** | Plasticizer |  | Plasticizer |  | |  |  |  |  |
| **Life Cycle Stage** | Use phase |  | Use phase |  | |  |  |  |  |
| **Exposed population(s)** | Children |  | Children |  | |  |  |  |  |
| **Product form** | Article |  | Article |  | | **0** |  |  |  |
| **Product delivery type** | N/A |  | N/A |  | | **0** |  |  |  |
| **Expected exposure route/use pattern** | Dermal, oral |  | Dermal, oral |  | | **0** |  |  |  |
| **Frequency/duration/amount of use** | ECETOC TRA, version 3.1 (June 2014) | ECETOC (2014) | ECETOC TRA, version 3.1 (June 2014) | ECETOC (2014) | | **0** | No difference | High |  |
|  | Amount: |  | Amount: |  |  |  |  |  |  |
| **Ingredient concentration in product** | Substitution factor = 1.00 | Grossman (2008) | Substitution factor = 1.03 | Grossman (2008) | | **0** | A little more is needed to produce same hardness level | High | High because based on measurements |
| **Ingredient total use volume** | Base |  | Base × 1.03 |  | | **0** | Assumes similar concentration and annual sales volume | High | High because based on measurements |
| **Other ingredients in the formula that may differentially impact potential for exposure** | N/A |  | N/A |  | | **0** |  | High |  |
| **Accessibility of ingredient in product and during use** | Bound in matrix |  | Bound in matrix |  | | **0** |  | High | High |
| **Separation potential during product life** | Potential for dust, oral extraction |  | Potential for dust, oral extraction |  | | **0** |  | High | Data gap |
| **Product disposal method** | Landfill |  | Landfill |  | | **0** |  | High | High |

N/A = not available.

**References**

[ECETOC] European Centre for Ecotoxicology and Toxicology of Chemicals. 2014. ECETOC TRA version 3.1 (June 2014) [Internet]. Auderghem (Belgium); European Centre for Ecotoxicology and Toxicology of Chemicals; [cited 6 October 2017]. Available from: http://www.ecetoc.org/tools/targeted-risk-assessment-tra/

Grossman R. 2008. Handbook of vinyl formulating. 2nd ed. Hoboken (NJ): John Wiley & Sons.

Haynes W. 1998. CRC handbook of chemistry and physics. 79th ed. Boca Raton (FL): CRC Press.

PBT Profiler. 2016. PBT Profiler (Persistent, Bioaccumulative, and Toxic Profiles Estimated for Organic Chemicals) [Internet]. [cited 6 October 2017]. Available from: http://www.pbtprofiler.net

SRC Inc. 2016. FatePointers Search Module (PhysProp) [Internet]. North Syracuse (NY): SRC Inc.; [cited 6 October 2017]. Available from: https://esc.syrres.com/fatepointer/webprop.asp?CAS=541913

[TOXNET] Toxicology Data Network. 2016. Hazardous Substances Data Bank (HSDB) (via the FatePointers Search Module) [Internet]. Bethesda (MD): US National Library of Medicine; [cited 6 October 2017]. Available from: http://toxnet.nlm.nih.gov/cgi-bin/sis/search2/f?./temp/~esQHvN:1

[USEPA] US Environmental Protection Agency. 2012. Estimation Program Interface (EPI Suite), version 4.11 [Internet]. Washington (DC): USEPA; [cited 6 October 2017]. Available from: https://www.epa.gov/tsca-screening-tools/epi-suitetm-estimation-

[USEPA] US Environmental Protection Agency. 2017. EPA ACToR physchemdb (qikprop), [Internet]. Washington (DC): USEPA; [cited 6 October 2017]. Available from: http://actorws.epa.gov/actorws/physchemdb/dev/properties

[USEPA] US Environmental Protection Agency. 2018. EPIWEB 4.1 [Internet]. Washington (DC): USEPA; [cited 03 April 2018]. Available from: https://www.epa.gov/tsca-screening-tools/download-epi-suitetm-estimation-program-interface-v411
